# Well-Defined pH-Sensitive Self-Assembled Triblock Copolymer-Based Crosslinked Micelles for Efficient Cancer Chemotherapy

**DOI:** 10.3390/molecules27238153

**Published:** 2022-11-23

**Authors:** Mohamed Alaa Mohamed, Ajay Singh, Paras N. Prasad, Chong Cheng

**Affiliations:** 1 Department of Chemical and Biological Engineering, University at Buffalo, The State University of New York, Buffalo, NY 14260, USA; 2 Chemistry Department, Faculty of Science, Mansoura University, Mansoura 35516, Egypt; 3 Institute for Lasers, Photonics and Biophotonics, Department of Chemistry, University at Buffalo, The State University of New York, Buffalo, NY 14260, USA

**Keywords:** ATRP, drug delivery, pH-responsive micelles, doxorubicin, cancer therapy

## Abstract

Delivery of chemotherapeutics to cancer cells using polymeric micelles is a promising strategy for cancer treatment. However, limited stability of micelles, premature drug release and off-target effect are the major obstacles that restrict the utilization of polymeric micelles as effective drug delivery systems. In this work, we addressed these issues through the innovative design of targeted pH-sensitive crosslinked polymeric micelles for chemotherapeutic delivery. A well-defined triblock copolymer, poly(ethylene glycol)-*b*-poly(2-hydroxyethyl methacrylate)-*b*-poly(butyl acrylate) (PEG-*b*-PHEMA-*b*-PBA), was synthesized by living radical polymerization, and then modified by using 4-pentenoic anhydride to incorporate pendant crosslinkable alkene groups in the middle block. The resulting copolymer underwent self-assembly in aqueous solution to form non-crosslinked micelles (NCMs). Subsequently, intramicellar thiol–ene crosslinking was performed by using 1,4-butanediol bis(3-mercaptopropionate) to give crosslinked micelles (CMs) with pH-sensitive crosslinks. The targeted CM (cRGD-DOX_10_-CM5) was readily prepared by using tumor-targeting ligand cyclo(Arg-Gly-Asp-D-Phe-Cys) (cRGD) together with the 1,4-butanediol bis(3-mercaptopropionate) during the crosslinking step. The study of cumulative DOX release revealed the pH-sensitive feature of drug release from these CMs. An in vitro MTT assay revealed that NCMs and CMs are biocompatible with MCF 10A cells, and the samples exhibited significant therapeutic efficiency as compared to free DOX. Cellular uptake studies confirmed higher uptake of cRGD-DOX_10_-CM5 by MCF 10A cancer cells via cRGD-receptor-mediated endocytosis as compared to the corresponding analogues without cRGD. These results indicate that such pH-responsive crosslinked PEG-*b*-PHEMA-*b*-PBA-based micelles are therapeutically effective against cancer cells and hold remarkable promise to act as smart drug delivery systems for cancer therapy.

## 1. Introduction

Although chemotherapy has become a cornerstone of cancer treatment, the efficacy of chemotherapeutic agents is still far from satisfaction due to the poor accumulation of drugs in the tumor sites, drug resistance, in vivo degradation and short plasma half-life time of drug, dose-limiting side effects and non-specific uptake by healthy tissues [1,2,3,4,5]. Drug delivery using nanoscopic carriers has emerged as a powerful approach to tackle these drawbacks of conventional chemotherapy, by avoiding rapid clearance, protecting drugs from enzymatic degradation, and bypassing efflux pumps [6]. Numerous drug carriers such as polymeric micelles [7], liposomes [8], nanogels [9], nanocapsules [10,11,12], and inorganic nanoparticles [13] have been investigated for the effective delivery of chemotherapeutics to cancer cells. Among all carriers, polymeric micelles with well-defined core-shell structures, formed by self-assembly of amphiphilic polymers in aqueous solutions, have attracted significant attention because of their passive tumor targeting ability through enhanced permeability and retention (EPR) effect, and ease of production with tailorable size and shape [14,15,16]. Moreover, the encapsulation of drugs within the hydrophobic micelle core promotes high drug doses without displaying an unacceptable level of adverse side effects [4,17]. In addition, hydrophilic coronas consisting of water-soluble polymers (e.g., polyethylene glycol (PEG)) ensure high stability of chemotherapeutics against external biological milieu and significantly reduce non-specific protein adsorption, resulting in prolonged circulation and bioavailability of the encapsulated anticancer drugs [18,19].

The major bottleneck that hinders the practical use of micelles in the delivery of therapeutics is their limited in vivo stability, which results from the dissociation of micelles upon the drop of polymer concentration below a critical micelle concentration (CMC) value because of excessive dilution with body fluids after intravenous injection [18,20,21]. Moreover, because of the poor structural integrity of micelles, the premature release of drugs before reaching the pathological sites and the leakage of drugs from the micelle cores during storage are unavoidable problems, which reduce the efficacy and shelf-life time of drug formulations [21,22]. Furthermore, relative to actively targeted micelles, passive targeted polymeric micelles usually encounter low tumor targeting ability, resulting in a low concentration of the drug in the tumor site and hence lower therapeutic efficiency [23,24,25,26].

Significant attempts have been made through chemical crosslinking of either core or shell of micelles to increase the stability of polymeric micelles and to prevent their dissociation, [27,28,29,30]. However, the utilization of stable covalent bonds for crosslinking of micelles not only significantly retards drug release, but also generates nano-carriers that cannot be readily eliminated from biological systems after drug release. Besides this, inappropriate choice of crosslinking chemistry would result in the participation of the drug in the crosslinking process and the potential loss of its therapeutic activity [31]. The dilemma between enhancing micelle stability while maintaining adequate drug release and elimination feasibility raises the need for the development of stimuli-sensitive cleavable micelles, which incorporate dynamic covalent bonds that undergo chemical cleavage in response to external stimuli [21]. Various types of cleavable linkages, such as acid labile groups [32], enzymatic cleavable moieties [33], redox-responsive groups [34], and photo-cleavable bonds [35], have been employed to develop stimuli-responsive micelles.

Among all stimuli-cleavable micelles, the development of pH-sensitive crosslinked micelles (CMs) has attracted significant attention because of the acidic environment of tumor tissues, and the feasibility of triggering the release of DOX by the acidic pH of endosomal-lysosomal compartments (pH 4.5–6) [34,36]. Several groups harnessed the acid-labile nature of hydrazone, ketal, acetal, cis-aconityl, and β-propionate linkages to develop either CMs or polymer-drug conjugates with pH-sensitivity [37,38,39,40,41,42]. Despite the success of the chemical crosslinking strategy in maintaining the structural integrity and keeping the micelles intact during blood circulation, the nature of chemical bonds and crosslinking degree critically influence the sizes of CMs, drug encapsulation efficiency, and drug release profile [21,43]. Excessive crosslinking of micelle core or shell would prevent rather than retard drug release. Also, the possible formation of inter-micellar crosslinking would significantly increase the particle size and reduce the stability and targeting ability of the micellar system. Thus, on the basis of the pioneering studies of CMs prepared by crosslinking of triblock copolymer micelles via reactive groups in the middle blocks, further research is needed to explore new designs of well-defined pH-sensitive CMs with nanoscopic dimensions and cleavable crosslinkages at core/shell intermediate layer to modulate drug release profile while achieving remarkable micelle stability.

**Scheme 1:**
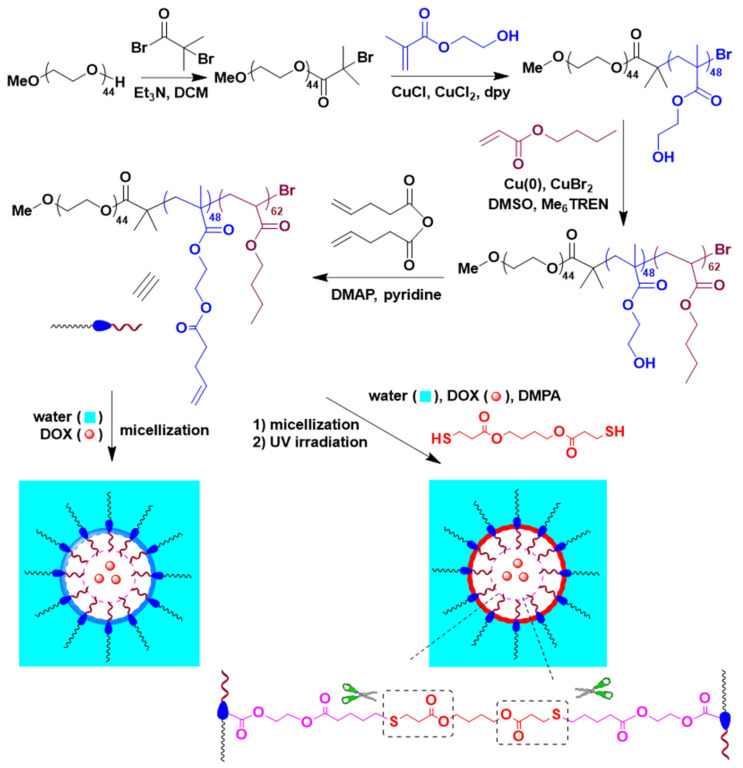
Synthesis of PEG-*b*-P(HEMA-ene)-*b*-PBA.

The aim of this work is to produce structurally stable pH-sensitive micelles yielding tunable DOX release with the potential for accumulation in tumor tissues. Toward this aim, targeted and pH-sensitive CMs were developed for selective DOX delivery in breast cancer (Scheme 1). Specifically, well-defined poly(ethylene glycol)-*b*-poly(2-hydroxyethyl methacrylate)-*b*-poly(butyl acrylate) (PEG-*b*-PHEMA-*b*-PBA) triblock copolymer was first synthesized through two consecutive living radical polymerization steps, followed by post modification of the pendant hydroxyl group of the middle poly(2-hydroxyethyl methacrylate) (PHEMA) block with 4-pentenoic anhydride to incorporate thiol-reactive alkene functionalities. Then, a series of DOX-loaded CMs with different crosslinking densities at the core/shell intermediate layer was achieved through a thiol-ene reaction using 1,4-butanediol bis(3-mercaptopropionate) as the pH-sensitive crosslinker and their drug release profile was evaluated. Also, targeted pH-responsive CMs were synthesized, in situ during crosslinking, through chemical conjugation of the thiol group of cysteine residue of cRGD to the pendant alkenes. Thereafter, in vitro bio-evaluations of these DOX-loaded micelles using MCF 10A breast cancer cells as representative cancer cells were investigated.

## 2. Result and Discussion

### 2.1. Synthesis and Characterization of PEG-b-P(HEMA-ene)-b-PBA

It is desirable for drug delivery systems to have a uniform size and prolonged stability at physiological conditions for the effective delivery of chemotherapeutics. In this work, living radical polymerization was employed to construct a well-defined PEG-*b*-PHEMA-*b*-PBA triblock copolymer, which was modified by using 4-pentenoic anhydride to give PEG-*b*-P(HEMA-ene)-*b*-PBA with pendant alkene groups attached to the middle block. The resulting modified triblock copolymer served as the building unit for the development of pH-sensitive CMs with tunable size, shape, morphology, and controlled crosslinking density at the core/shell intermediate layer.

**Figure 1:**
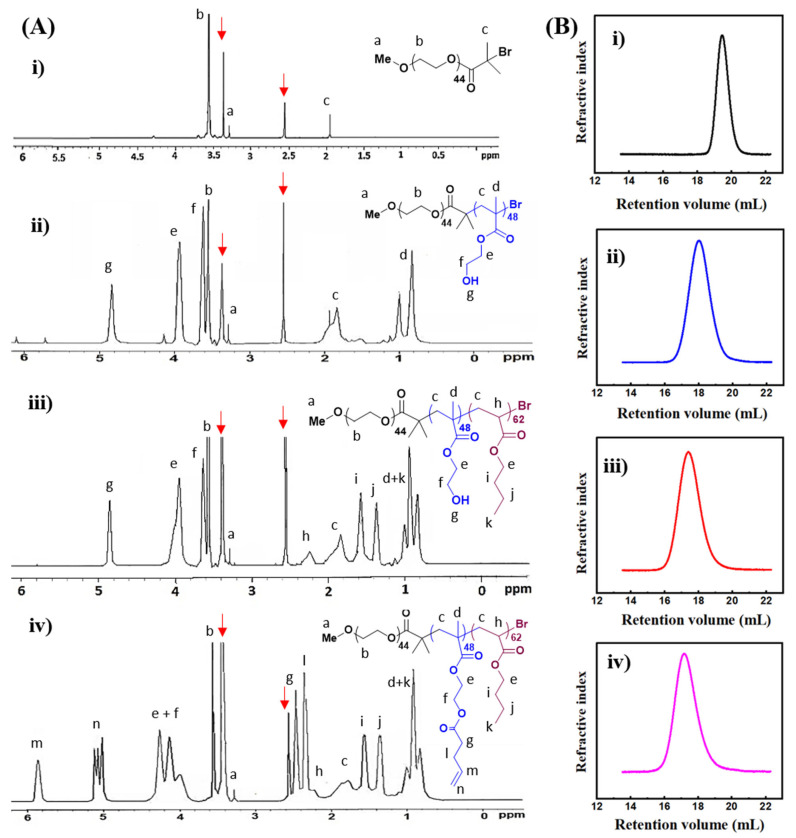
(**A**) ^1^H NMR spectra and (**B**) GPC analysis of (**i**) PEG-macroinitiator; (**ii**) PEG-*b*-PHEMA; (**iii**) PEG-*b*-PHEMA-*b*-PBA; and (**iv**) PEG-*b*-P(HEMA-ene)-*b*-PBA in DMSO-d_6_. Resonances from solvent are indicated with red arrows.

Synthesis of PEG-*b*-P(HEMA-ene)-*b*-PBA is described in Scheme 1. MeO-PEG-Br macroinitiator was first prepared through a reaction of MeO-PEG-OH with 2-bromo-2-methylpropionyl bromide in presence of Et_3_N and then used to access triblock polymeric structure via living radical polymerization. The chemical structure of the MeO-PEG-Br macroinitiator was confirmed by ^1^H NMR analysis, which revealed all characteristic resonances corresponding to the protons from the target chemical structure (Figure 1A(i)). Importantly, the ratio of resonance intensity of terminal methoxyl protons at 3.28 ppm to that of methyl protons of bromoester terminal at 1.94 ppm was 1/2, confirming the complete conversion of OH terminal of MeO-PEG-OH during the reaction. Number-average polymerization (*DP*_n_) of 44 was calculated from the integration ratio between the EG protons (OC*H*_2_) at 3.55 ppm and the OC*H*_3_ protons at 3.28 ppm, and the number-average MW (*M*_n_) obtained from NMR was 2.3 kDa. GPC trace of MeO-PEG-Br macroinitiator showed a monomodal elution peak of *M*_n_ of 2.17 kDa and MW dispersity (*Ð*) of 1.01, relative to linear polystyrene (Figure 1B(i); Table 1).

Subsequently, PEG-*b*-PHEMA-*b*-PBA triblock copolymer was synthesized in two successive living radical polymerization steps. Firstly, the diblock copolymer of PEG-*b*-PHEMA was synthesized through ATRP of HEMA using MeO-PEG-Br macroinitiator, in the presence of CuCl/dpy as the activating complex and CuCl_2_ as the deactivator. Secondly, chain extension of PEG-*b*-PHEMA diblock copolymer with BA was conducted using Cu(0)-mediated radical polymerization. The resulting diblock and triblock copolymers, PEG-*b*-PHEMA and PEG-*b*-PHEMA-*b*-PBA, were characterized by ^1^H NMR and GPC analysis. Their ^1^H NMR spectra were shown in Figure 1A(ii,iii), with characteristic resonances from each block clearly assigned. Based upon the comparison of the resonance intensities of characteristic protons from EG units at 3.55 ppm (OC*H*_2_), HEMA units at 3.93 ppm (OCOC*H*_2_, for diblock copolymer) and BA units at 2.24 ppm (C*H*, for triblock copolymer).

*DP*_n_ of 62 for PHEMA and 48 for PBA blocks, as well as the *M*_n_ of 10.2 kDa for PEG-*b*-PHEMA and 16.4 kDa for PEG-*b*-PHEMA-*b*-PBA, were obtained by ^1^H NMR analysis (Table 1). Therefore, the accurate structure of the triblock copolymer can be expressed as PEG_44_-*b*-PHEMA_48_-*b*-PBA_62_. GPC traces of PEG-*b*-PHEMA and PEG-*b*-PHEMA-*b*-PBA are shown in Figure 1B(ii,iii). These copolymers demonstrated monomodal elution peaks, with an obvious shift to high MW sides along with chain extension. GPC analysis showed *M*_n_ of 10.4 and *Ð* of 1.15 for PEG-*b*-PHEMA, and *M*_n_ of 16.7 and *Ð* of 1.21 for PEG-*b*-PHEMA-*b*-PBA, relative to linear polystyrene. Such GPC results confirmed well-controlled chain growth via the living radical polymerization process.

Post-polymerization functionalization of PEG-*b*-PHEMA-*b*-PBA was performed by treating the triblock copolymer with 4-pentenoic anhydride in the presence of pyridine, in order to incorporate thiol-reactive alkene functionalities with the middle PHEMA block. ^1^H NMR spectrum of the resulting PEG-*b*-P(HEMA-ene)-*b*-PBA is shown in Figure 1A(iv). The appearance of characteristic resonances of alkene protons at around 5.02 and 5.82 ppm, together with the disappearance of characteristic hydroxyl proton resonances of PHEMA block at 4.80 ppm and the shift of methylene proton adjacent to the hydroxyl group of PHEMA from 3.62 to 3.80–4.20 ppm, confirmed the complete conversion of HEMA units to alkene-functionalized HEMA-ene units. The GPC trace of PEG-*b*-P(HEMA-ene)-*b*-PBA is shown in Figure 1B(iv), with a significant shift to the high MW side as compared to that of PEG-*b*-PHEMA-*b*-PBA. According to GPC analysis, it has an M_n_ of 22.0 kDa and a *Ð* of 1.27 relative to linear polystyrene.

### 2.2. Preparation of PEG-b-P(HEMA-ene)-b-PBA NCMs and CMs

It is well-demonstrated that amphiphilic block copolymers self-assemble into micelles in certain solvents when their concentrations are above CMC. To investigate the CMC of triblock PEG-*b*-P(HEMA-ene)-*b*-PBA in aqueous solutions, analysis by fluorescence spectroscopy using the pyrene probe was utilized. Fluorescence spectra of pyrene were recorded at room temperature in presence of an increasing concentration of PEG-*b*-P(HEMA-ene)-*b*-PBA (Figure S1A). Following a literature approach [44], the fluorescence intensity at 373 nm (I_373_), instead of the intensity ratio I_373_/I_384_, was used for CMC determination because of the overlap between emission peaks of pyrene. As a result, a CMC value of 16 mg/L was obtained for PEG-*b*-P(HEMA-ene)-*b*-PBA from the intersection of two lines in the I_373_ versus the Log C chart (Figure S1B). The low CMC value of PEG-*b*-P(HEMA-ene)-*b*-PBA triblock copolymer can be attributed to the pronounced hydrophobicity of PBA block and the P(HEMA-ene) middle block relative to the PEG block, although the P(HEMA-ene) block is still less hydrophobic than the PBA block.

PEG-*b*-P(HEMA-ene)-*b*-PBA NCMs were prepared by adding DMSO solutions of the triblock PEG-*b*-P(HEMA-ene)-*b*-PBA copolymer at a concentration of 10 mg/mL to the aqueous solution under vigorous stirring. With its amphiphilic nature, it self-assembled into three layered micelles with a core domain, core/shell intermediate layer, and an outer shell consisting of highly hydrophobic PBA block, moderately hydrophobic P(HEMA-ene), and hydrophilic PEG block, respectively. The core/shell intermediate layer carries pendant alkene groups, which can serve as chemical handles for further crosslinking reactions.

**Figure 2:**
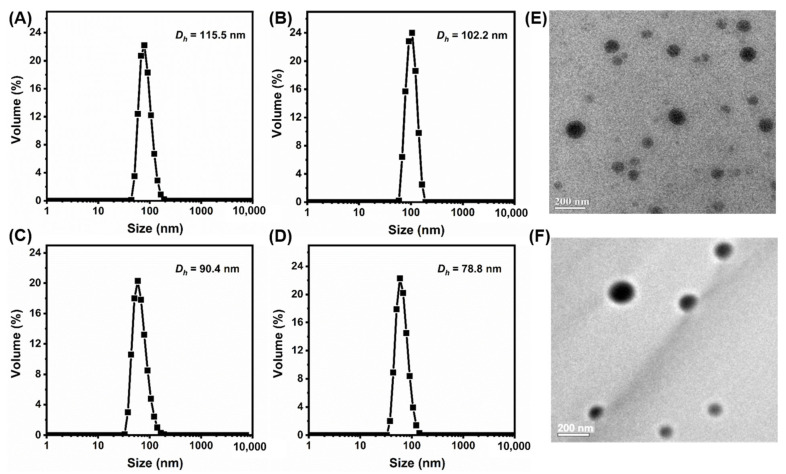
DLS diagrams of PEG-*b*-P(HEMA-ene)-*b*-PBA-based nanoparticles: (**A**) NCM, (**B**) CM5, (**C**) CM10, and (**D**) CM20 in water. TEM images of (**E**) NCM and (**F**) NCM5.

PEG-*b*-P(HEMA-ene)-*b*-PBA CM samples were prepared by using thiol-ene chemistry (Scheme 1). 1,4-Butanediol bis(3-mercaptopropionate) was used to crosslink the alkene-containing core-shell intermediate micelle layer, with the formation of pH-sensitive crosslinkages. The choice of fast, orthogonal thiol-ene reaction for crosslinking provides additional advantage to the synthetic design because the impact of crosslinking on the colloidal stability of micelles is minimized and harmful byproducts are avoided. A series of crosslinked PEG-*b*-P(HEMA-ene)-*b*-PBA micelles were prepared through UV irradiation of the reaction systems, with different [thiol]/[ene] molar feed ratios (5, 10, and 20%) in presence of DMPA as photoinitiator (Table 2). These samples are denoted as CM5, CM10, and CM20, where the number refers to the molar percentage of the thiol group of crosslinker relative to the alkene functionality of the polymer. DLS analysis of NCM, CM5, CM10, and CM20 samples revealed their volume-average hydrodynamic diameters (*D*_h,v_) of 115.5 ± 4.4, 102.2 ± 2.4, 90.4 ± 2.3, and 78.8 ± 1.6 nm, respectively, with narrow size distribution (Figure 2A–D). It was noticeable that *D*_h,v_ significantly decreased with the increase of the [thiol]/[ene] molar feed ratio from 5 to 20%. This trend can be attributed to the formation of compact micelles upon crosslinking the pendant alkene groups of the intermediate micelle layer, as well as the more restricted swelling of micelles with the increase of crosslinking degree. TEM imaging was also utilized to characterize NCMs and CMs (Figure 2E,F and Figure S2). Their sizes on TEM grids are in the same magnitude as their hydrodynamic sizes by DLS. Similar to NCMs, CMs also exhibited spherical morphologies, indicating that crosslinking occurred only within micelles.

### 2.3. Preparation of DOX-Loaded PEG-b-P(HEMA-ene)-b-PBA NCMs and CMs

DOX_10_-CM5 and DOX_10_-CM10 were readily prepared by adding DMSO solutions of DOX (10 wt% relative to polymer), triblock PEG-*b*-P(HEMA-ene)-*b*-PBA copolymer, 1,4-butanediol bis(3-mercaptopropionate) crosslinker (with 5 or 10 mol% of thiol relative to alkene of the polymer), and DMPA to aqueous solutions of pH 7.4, followed by UV irradiation for 30 min, and subsequently dialyzed against PBS at pH 7.4 to remove free DOX. Similarly, targeted cRGD-conjugated DOX-loaded crosslinked micelles (cRGD-DOX_10_-CM5) were prepared by direct reaction of thiol-containing RGD with the P(HEMA-ene) middle block during the integrated step of crosslinking and DOX loading. DOX-loaded NCMs (DOX_7_-NCM and DOX_10_-NCM) were prepared by the same approach, using two different concentrations of DOX in the feed (7 and 10 wt% relative to polymer, respectively), but without adding both crosslinker and DMPA. Preparation conditions and DLS analytic results of these DOX-loaded micelles are shown in Table 3. Relative to blank micelles, DOX-loaded CM and NCM samples have noticeable increases in hydrodynamic size due to the presence of encapsulated DOX. For instance, *D*_h,v_ of DOX_10_-NCM was 128.3 ± 3.4 nm (PDI = 0.24), which was considerably larger than *D*_h,v_ of 115.5 ± 4.4 nm (PDI = 0.20) for DOX-free NCM. Interestingly, after crosslinking using 5 and 10 mol% of thiol relative to alkene, *D*_h,v_ of the resulting micelles gradually decreased to 106.9 ± 1.1 nm (PDI = 0.11) and 95.1 ± 2.4 nm (PDI = 0.10), respectively. The large *D*_h,v_ of cRGD-DOX_10_-CM5 (135.5 ± 4.4 nm) relative to the DOX_10_-CM5 sample may have resulted from the successful conjugation of cRGD-SH to the micelles. For the DOX-loaded NCMs, the DOX loading amount increased from 6.2 to 9.1 wt% with the increase of the feeding amount of DOX from 7 to 10 wt% relative to the polymer. Of note, designing drug carriers with high loading capacity is critical for efficient cancer therapy. Therefore, we decided to proceed with the higher DOX wt%, and subsequently, the micelles were crosslinked to maintain high DOX loading. It is worth noting that at the same feeding concentration of DOX (10 wt% relative to polymer), DOX loading efficiency increased from 91 to 97% by the presence of 5% crosslinking of the micellar core-shell intermediate layer, indicating that crosslinking can increase the resistance for the diffusion of DOX from the micelle cores during dialysis step and therefore promote the encapsulation efficiency.

### 2.4. In Vitro pH-Responsive DOX Release

**Figure 3:**
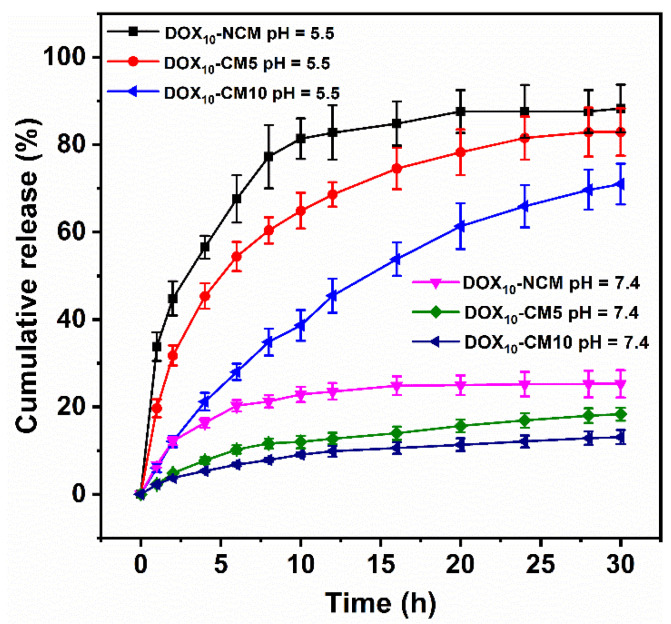
In vitro DOX release profiles from DOX-loaded PEG-*b*-P(HEMA-ene)-*b*-PBA micelles in PBS buffer at pH 5.5 and 7.4 at 37 °C.

To demonstrate the pH-sensitivity of PEG-*b*-P(HEMA-ene)-*b*-PBA based CMs, DOX release from DOX_10_-NCM, DOX_10_-CM5, and DOX_10_-CM10 samples were investigated at two different pH conditions (pH 5.5 and 7.4) using dialysis method (Figure 3). Limited DOX release was noticed at pH 7.4 for all samples. Specifically, after 2 h of incubation, DOX_10_-NCM, DOX_10_-CM5, and DOX_10_-CM10 displayed 12.2, 4.8, and 3.7% release of DOX, respectively. The slow release at pH 7.4 can be interpreted based on the hydrophobic nature of DOX at a relatively high pH, which increased the hydrophobic interaction between DOX and the PBA core. Moreover, for DOX_10_-CM5 and DOX_10_-CM10 samples, the existence of intact crosslinked core/shell intermediate layer retarded diffusion of DOX from micelles core. Previous work by Liu et al. ascribes a limited release of DOX at pH 7.4 for the same reason [45].

In contrast, at pH 5.5, the DOX_10_-NCM sample showed an initial burst release of 44.8% after 2 h. Within the same time period, DOX_10_-CM5 and DOX_10_-CM10 samples released 31.7 and 12.1% of DOX, respectively. The fast DOX release from DOX_10_-NCM at pH 5.5 can be partly attributed to the increase of hydrophilicity of DOX at lower pH 5.5, and hence higher solubility of DOX at low pH medium and rapid diffusion outside the NCM core. Importantly, cleavage of pH-sensitive crosslinkages at the core/shell intermediate layer of DOX_10_-CM5 and DOX_10_-CM10 samples significantly promoted DOX release at pH 5.5 (6.6 and 3.2-fold increase in released dose) as compared to the release at pH 7.4 from the same samples. Additionally, the decrease of DOX release rate with increasing the crosslinking [thiol]/[ene] molar ratio at the core/shell intermediate layer, was in line with our proposed hypothesis and demonstrated the feasibility of regulation of DOX release through controlling the permeability of crosslinked core-shell intermediate layer. The pH-sensitive release of DOX makes the system promising for intracellular drug delivery, in which DOX release would be stimulated by the low pH of late endosomes.

### 2.5. In Vitro Cytotoxicity

**Figure 4:**
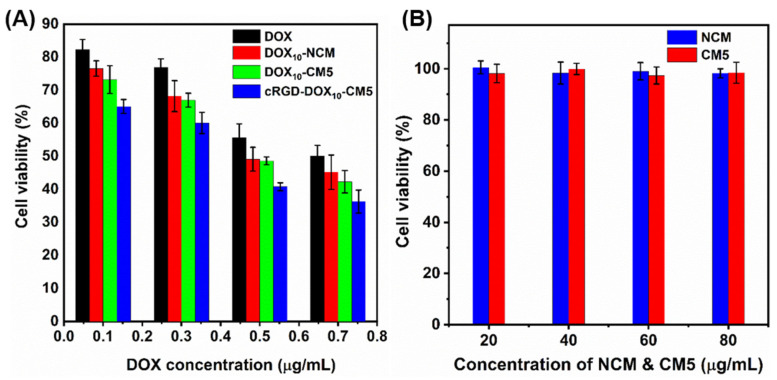
Cytotoxicity of (**A**) DOX, DOX_10_-NCM, DOX_10_-CM5, cRGD-DOX_10_-CM5, and (**B**) DOX-free NCM and CM5 samples against MCF 10A breast cancer cells after 24 h of incubation.

The in vitro cytotoxicity of DOX_10_-NCM, DOX_10_-CM5, and cRGD-DOX_10_-CM5 samples was evaluated by the MTT assay against MCF 10A cancer cell line at the DOX concentration range of 0.1–0.7 μg/mL (Figure 4A). Free-DOX was used as the positive control, while blank NCM and CM5 were used as the negative control. cRGD-DOX_10_-CM5 exhibited a higher therapeutic efficiency (IC_50_ ~0.4 μg/mL) after 24 h as compared to DOX_10_-NCM (IC_50_ ~0.48 μg/mL) and DOX_10_-CM5 (IC_50_ ~0.48 μg/mL) at the same DOX concentration; this is most likely due to higher cellular uptake of cRGD-DOX_10_-CM5 by RGD-receptor-mediated endocytosis, which can be explained through selective recognition of cRGD by α_v_β_3_ and α_v_β_5_ integrin expressed on tumor cells [46]. Moreover, there was no significant variation in the cytotoxicity of DOX_10_-NCM2 and DOX_10_-CM5. However, both samples displayed higher therapeutic activity relative to free DOX, presumably because of the synergistic role of the polymeric carriers in enhancing cellular uptake. Relative to DOX-loaded samples, NCM and CM5 showed no considerable cytotoxicity and higher cell viability was maintained after incubation with 80 μg/mL of these scaffolds for 24 h (Figure 4B). Taken together, the results suggested that cRGD-DOX_10_-CM5 can be promising in cancer treatment without encountering adverse cytotoxic effects.

### 2.6. Cellular Uptake

**Figure 5:**
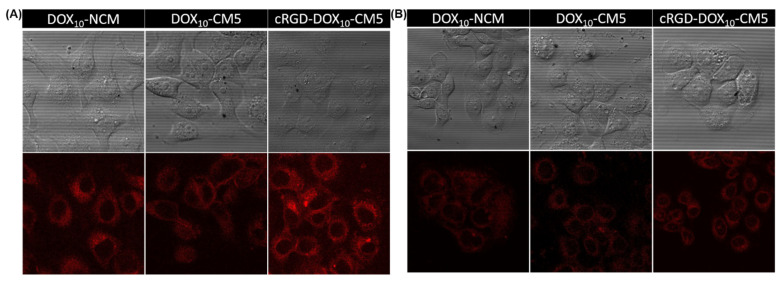
CLSM images of (**A**) MCF 10A cancer cells, and (**B**) MCF 10A normal cells after 2 h incubation with DOX_10_-NCM, DOX_10_-CM5, cRGD-DOX_10_-CM5.

To verify the effect of targeting ligands on internalization and cellular uptake efficiency, cRGD- DOX_10_-CM5 was incubated with the cRGD receptor-positive MCF 10A cancer cell for 2 h and then examined using confocal laser scanning microscopy (CLSM) by probing DOX fluorescence. The result was compared with cRGD-free DOX_10_-NCM and DOX_10_-CM5 as negative controls. As shown in Figure 5A, it is obvious that cRGD-DOX_10_-CM5 displayed higher DOX fluorescence intensity as compared to DOX_10_-NCM and DOX_10_-CM5 samples. This result indicates substantial cellular internalization into the cytoplasm of MCF 10A cancer cells by cRGD-receptor-mediated endocytosis. For further confirmation of the promoted internalization by cRGD-mediated endocytic pathway, an additional cellular uptake study was investigated using a cRGD-receptor negative MCF 10A normal cell line. The resulting CLSM images showed insignificant differences in the DOX fluorescence intensity of cRGD-DOX_10_-CM5 compared to DOX_10_-NCM and DOX_10_-CM5 (Figure 5B). Overall, the results reveal that the cRGD-DOX_10_-CM5 could specifically and efficiently deliver DOX to cancer cells via receptor-mediated binding and intracellular uptake, which may lead to enhanced therapeutic efficacy to targeted cancer cells.

## 3. Discussion

pH-Sensitive crosslinked micelles with enhanced stability and prolonged circulation in the blood stream were developed for effective delivery of DOX to breast cancer cells. A well-defined PEG-*b*-PHEMA-*b*-PBA triblock copolymer was synthesized by two ATRP steps from the PEG-Br macroinitiator. ATRP technique was selected to control the chain growth and block length, and to achieve narrow molecular weight dispersity [47,48]. PEG was used as a hydrophilic polymer to stabilize the resultant structure and it has been used in numerous FDA-approved products [49,50]. PBA block was used to confer the structure with hydrophobicity that enables self-assembly as well as promotes the interaction with the hydrophobic DOX, thereby increasing the drug loading capacity. It is worth mentioning that the molecular weight of the final polymer was maintained below the renal filtration threshold (~45 kDa) to allow clearance from the body after releasing the payload [51]. The triblock copolymer was modified at the middle PHEMA block by reacting the OH groups with 4-pentenoic anhydride to install photoreactive olefin groups to the polymer structure. The resultant PEG-*b*-P(HEMA-ene)-*b*-PBA triblock copolymer self-assembled in aqueous solutions to form NCM, with PBA at the core and PEG at the shell. Further crosslinking of the pendant olefin groups using pH-sensitive 1,4-butanediol bis(3-mercaptopropionate) could stabilize the structure and tune the release behavior of DOX. Thiol-ene crosslinking was selected because of the fast reaction kinetics, without generating toxic by-products [52]. By varying the crosslinking density through changing the [SH]/[ENE] percent (5, 10, 20 mol%), a series of CMs were obtained. DLS measurements of NCMs and CMs revealed a decrease in particle sizes as a result of crosslinking. This behavior was attributed to the formation of compact micelles and the restricted swelling of the structures. For DOX-loaded CMs, the cumulative DOX release was inversely correlated with crosslinking density due to the restricted diffusion of DOX with crosslinking. In vitro cytotoxicity of the DOX-free CMs against MCF 10A revealed their biocompatibility. cRGD was used as a target ligand with affinity to α_v_β_3_ and α_v_β_5_ integrins expressed on cancer cells [53,54]. Cellular uptake study revealed higher DOX fluorescence intensity from MCF 10A cells treated with cRGD-DOX_10_-CM5 compared with those treated with DOX_10_-NCM and DOX_10_-CM5, due to receptor-mediated endocytosis. Overall, this study suggests that pH-sensitive self-assembled nanoparticles with tunable sizes, crosslinking density, controlled drug release, and efficient cellular uptake are promising for cancer therapy.

## 4. Materials and Methods

### 4.1. Chemicals

2-Hydroxyethyl methacrylate (HEMA, 99%), butyl acrylate (BA, 99%), poly(ethylene glycol)methyl ether (MeO-PEG-OH, MW = 2000 Da, 99%), 4-pentenoic anhydride (98%), 4-(dimethylamino)pyridine (DMAP, 99%), 2,2-diphenyl-1-picrylhydrazyl (DPPH), copper (Ⅰ) chloride (CuCl, 99%), copper (Ⅱ) chloride (CuCl_2_, 99%), basic and neutral alumina, 3-[4,5-dimethylthiazol-2-yl]-2,5-diphenyl tetrazoliumbromide (MTT), fetal bovine serum (FBS), and PBS buffers were purchased from Sigma-Aldrich (St. Luis, MO, USA). 2-Bromoisobutyryl bromide (98%), 2,2’-dibyridyl (dpy, 99%), 2,2-dimethoxy-2-phenylacetophenone (DMPA, 99+%) and pyrene (98%) were purchased from Acros Organics (Fair Lawn, NJ, USA). Dichloromethane (DCM), dimethyl sulfoxide (DMSO), tetrahydrofuran (THF), tris [2-(dimethylamino)ethyl]amine (Me_6_TREN, 97%), triethylamine (Et_3_N, 99%), pyridine (99%) and bare Cu(0) wire (20 AWG) were purchased from Fisher Scientific (Hampton, NH, USA). 1,4-Butanediol bis(3-mercaptopropionate) (98%) was obtained from TCI (Portland, OR, USA). Dulbecco’s Modified Eagle Medium (DMEM) was purchased from Life Technologies (Carlsbad, CA, USA). Doxorubicin hydrochloride salt (DOX.HCl, 99%) was obtained from LC laboratories (Woburn, MA, USA). Cyclo(Arg-Gly-Asp-D-Phe-Cys) (cRGD) was purchased from Bachem Co (Torrance, CA, USA).

HEMA was purified following a literature approach [55]. BA was passed through a column of basic alumina prior to use. DCM and DMSO were dried by distillation over CaH_2_. THF was dried by refluxing over sodium-benzophenone for 24 h before distillation. Cu(0) wire was activated following a literature method [56]. Other chemicals and reagents were used as received unless otherwise stated.

### 4.2. Measurements

^1^H NMR spectra were recorded using a Varian INOVA-500 spectrometer (Varian, Palo Alto, CA, USA) at room temperature, and the samples were dissolved either in DMSO-d_6_ or CDCl_3_ containing 1.0 vol% tetramethylsilane (TMS) as internal standard. Gel permeation chromatography (GPC) analysis of polymers was performed using a Viscotec GPC system (Malvern Ltd., Malvern, UK) equipped with a VE-3580 refractive index (RI) detector. DMF with 0.1 M LiBr was used as eluent for GPC with a flow rate of 0.5 mL/min at 55 °C. Monodispersed polystyrene standards of different molecular weights (MWs), purchased from Varian, were used to calibrate the GPC instrument. Dynamic light scattering (DLS) measurements were performed using a Nano ZS90 zetasizer (Malvern Instruments, Malvern, UK). A 4 mW 633 nm He Ne laser was used as the light source. All measurements were performed in an aqueous system at room temperature and a measurement angle of 90° to the incident laser beam. The effective diameter (D_h_) was calculated from the average of five measurements on each sample. Transmission electron microscope (TEM) images were obtained using a JEOL 2010 microscope (JEOL, Tokyo, Japan). TEM samples were prepared by dip coating 300 mesh carbon coating copper grid in a diluted solution (1.0 mg/mL) of CMs or non-crosslinked micelles (NCMs).

Steady-state fluorescence spectra were obtained using a PTI fluorescence spectrophotometer from Photon Technology International (Birmingham, NJ, USA). The excitation and emission slit widths were 5 nm, and the emission spectra were scanned from 340 to 450 nm with an excitation wavelength of 334 nm. UV/Vis spectra were recorded using a ThermoSpectronic (model 4001/4) spectrophotometer (Thermo Fisher Scientific, Waltham, MA, USA). For photoluminescence imaging of cells, a laser scanning confocal microscope (Leica, Wetzler, Germany) was used.

### 4.3. Synthesis of MeO-PEG-Br Macroinitiator

In a 250-mL round bottom flask, MeO-PEG-OH (2.25 g, 1.12 mmol, 1.0 equiv), Et_3_N (0.23 g, 2.24 mmol, 2.0 equiv), and DCM (10 mL) were added. The reaction mixture was stirred in an ice bath and 2-bromoisobutyryl bromide (0.52 g, 2.25 mmol, 2.0 equiv) was added dropwise using a syringe pump. Subsequently, the reaction was allowed to proceed at room temperature for 24 h. The reaction mixture was filtered to remove the triethylammonium bromide salt, then diluted with DCM (200 mL), and washed three times with HCl aqueous solution (1.0 M), three times with NaHCO_3_ aqueous solution (5% *w*/*v*) and two times with brine. The DCM layer was dried over anhydrous Na_2_SO_4_, and the volume was reduced to 10 mL by evaporating using a rotary evaporator. The concentrated DCM solution was precipitated in diethyl ether. The precipitant was collected by filtration and dried overnight under vacuum, to give MeO-PEG-Br macroinitiator as a white solid (1.76 g; 73% yield). ^1^H NMR (500 MHz, DMSO-d_6_, ppm) δ: 1.94 (s, C(C*H*_3_)_2_), 3.28 (s, C*H*_3_O), 3.55 (m, C*H*_2_O from EG).

### 4.4. Synthesis of MeO-PEG-b-PHEMA Diblock Copolymer

MeO-PEG-*b*-PHEMA macroinitiator was synthesized via atom transfer radical polymerization (ATRP) of HEMA using MeO-PEG-Br as a macroinitiator. In a 50-mL pre-flamed Schlenk flask equipped with a magnetic stirring bar, were added HEMA (6.50 g, 50 mmol, 200 equiv), MeO-PEG-Br (0.50 g, 0.25 mmol, 1.0 equiv), dpy (78.0 mg, 0.50 mmol, 2.0 equiv), CuCl_2_ (2.03 mg, 0.015 mmol, 0.06 equiv), and MeOH/2-butanone (3:2 *v*/*v*, 6.5 mL). The reaction flask was flushed with N_2_ and sealed with a rubber septum. The reaction mixture was stirred until all MeO-PEG-Br was dissolved, then degassed by five cycles of freeze-pump-thaw process. During the last cycle, CuCl (23.26 mg, 0.23 mmol, 0.94 equiv) was added very quickly to the frozen mixture, under a positive flow of N_2_. The system was evacuated and backfilled with N_2_ five times, and the flask was placed in an oil bath at 40 °C to start the reaction. After 2 h, the polymerization reaction was stopped by bubbling the solution with air. The reaction mixture was diluted with THF and passed through a column of neutral alumina to remove the copper complex. The eluted solution was concentrated under reduced pressure and then precipitated from diethyl ether. The precipitant was collected by filtration and dried under vacuum, to give MeO-PEG-*b*-PHEMA diblock copolymer (2.19 g, 31% yield). ^1^H NMR (500 MHz, DMSO-d_6_, ppm) δ: 0.72–1.18 (br m, C*H*_3_ from HEMA and C(C*H*_3_)_2_), 1.44–2.10 (br m, C*H*_2_ from HEMA), 3.28 (s, C*H*_3_O), 3.55 (m, C*H*_2_O from EG), 3.62 (m, C*H*_2_OH from HEMA), 3.93 (m, OCOC*H*_2_ from HEMA), 4.84 (br s, O*H* from HEMA).

### 4.5. Synthesis of MeO-PEG-b-PHEMA-b-PBA Triblock Copolymer

Linear MeO-PEG-*b*-PHEMA-*b*-PBA was prepared by Cu(0)-mediated radical polymerization of BA using MeO-PEG-*b*-PHEMA-Br macroinitiator. BA (1.25 g, 9.78 mmol, 100 equiv), MeO-PEG-*b*-PHEMA (1.0 g, 0.097 mmol, 1.0 equiv), CuBr_2_ (4.36 mg, 0.0195 mmol, 0.2 equiv), and Me_6_TREN (6.75 mg, 0.0293 mmol, 0.3 equiv) were added into a 50-mL Schlenk flask. Then, DMSO (1.25 mL) was added, and the reaction mixture was stirred until the complete dissolution of all solids. After five cycles of the freeze-pump-thaw process, 2.5 cm of activated Cu(0) wire was added to the frozen solution under a high stream of N_2_. The flask was evacuated and refilled with N_2_ three times, then placed in an oil bath at 30 °C. After 3 h, the polymerization was terminated by opening the flask to air and removing the copper wire. The polymerization solution was diluted with THF and then passed through a column of neutral alumina. Afterward, the eluted solution was dialyzed against MeOH for 3 days using a dialysis tubing with an MW cut-off (MWCO) of 3500 Da. The resulting solution was concentrated and then precipitated in hexane. The precipitant was collected by filtration and dried under vacuum, to give the MeO-PEG-*b*-PHEMA-*b*-PBA triblock copolymer (1.58 g, 70% yield). ^1^H NMR (500 MHz, DMSO-d_6_, ppm) δ: 0.72–1.18 (br m, C*H*_3_ from HEMA, BA, and C(C*H*_3_)_2_), 1.35–2.10 (br m, C*H*_2_ from HEMA and BA), 2.24 (br m, C*H* from BA), 3.28 (s, C*H*_3_O), 3.55 (m, C*H*_2_O from EG), 3.62 (m, C*H*_2_OH from HEMA), 3.80–4.20 (br m, OCOC*H*_2_ from HEMA and BA), 4.84 (br s, O*H* from HEMA).

### 4.6. Synthesis of MeO-PEG-b-P(HEMA-ene)-b-PBA

MeO-PEG-*b*-P(HEMA-ene)-*b*-PBA, a triblock copolymer with an alkene-functionalized middle block, was prepared by esterification reaction of the middle PHEMA block of MeO-PEG-*b*-PHEMA-*b*-PBA. In a typical experiment, a mixture of MeO-PEG-*b*-PHEMA-*b*-PBA (0.62 g, with 2.44 mmol of OH, 1.0 equiv), 4-pentenoic anhydride (2.22 g, 12.2 mmole, 5.0 equiv), DMAP (0.059 g, 0.49 mmol, 0.2 equiv) and pyridine (0.97 g, 12.2 mmol, 5.0 equiv) in DMF (5 mL) was stirred for 24 h at room temperature. Then the reaction solution was dialyzed against acetone for 1 week using dialysis tubing (MWCO = 3500 Da). The resulting solution was dried under vacuum to give MeO-PEG-*b*-P(HEM-ene)-*b*-PBA (0.93 g, 87% yield). ^1^H NMR (500 MHz, DMSO-d_6_, ppm) δ: 0.70–1.20 (br m, C*H*_3_ from HEMA-ene, BA and C(C*H*_3_)_2_), 1.30–2.10 (br m, C*H*_2_ from HEMA-ene and BA), 2.12–2.50 (br m, O_2_CC*H*_2_C*H*_2_CH= from PHMA-ene and C*H* from BA), 3.28 (s, C*H*_3_O), 3.55 (m, C*H*_2_O from EG), 3.62 (m, C*H*_2_OH from HEMA), 3.80–4.20 (br m, OCOC*H*_2_C*H*_2_OCO from HEMA-ene and OCOC*H*_2_ from BA), 4.95–5.18 (m, C*H*_2_=CH from HEMA-ene), and 5.82 (br, CH_2_=C*H* from HEMA-ene).

### 4.7. Determination of CMC of MeO-PEG-b-P(HEMA-ene)-b-PBA

CMC of MeO-PEG-*b*-P(HEMA-ene)-*b*-PBA was determined using pyrene as a fluorescent probe. In a typical experiment, a stock solution of pyrene (6.0 × 10^−5^ M) in acetone was prepared, and then a fixed volume of the solution was added to a series of vials followed by evaporation of acetone under reduced pressure. Then, different amounts of an aqueous micellar solution of MeO-PEG-*b*-P(HEMA-ene)-*b*-PBA, at the concentration of 1.0 mg/mL, was added to each vial, followed by dilution with deionized water to reach a final concentration of pyrene of 6 × 10^−7^ M and the final polymer concentrations of 0.1 to 64 mg/mL. All vials were incubated in dark for 6 h, and the fluorescence spectra were measured. The emission spectra were scanned from 340–450 nm at a fixed excitation wavelength of 334 nm. CMC was determined based on the fluorescence intensity at 373 nm.

### 4.8. Preparation of Blank and DOX-Loaded MeO-PEG-b-P(HEMA-ene)-b-PBA NCMs

Blank NCM was prepared by adding 2 mL of MeO-PEG-*b*-P(HEMA-ene)-*b*-PBA solution in DMSO (5 mg/mL) dropwise to 3 mL of deionized water. The micellar solution was stirred for 3 h then transferred to a dialysis bag (MWCO = 3500 Da) and dialyzed against deionized water for 24 h. For DLS measurements, the solution was diluted to 1 mg/mL.

For the preparation of DOX-loaded NCMs, a stock solution of DOX.HCl in DMSO (50.0 mg/mL) was first prepared and neutralized with Et_3_N (2 equiv). Different aliquots of the DOX solution were added to 2 mL of DMSO containing 10 mg of MeO-PEG-*b*-P(HEMA-ene)-*b*-PBA, with 7 and 10 wt% of DOX relative to the polymer in the feed. Then, the solution was added slowly to 3 mL of deionized water with stirring at 700 rpm for 3 h. The final solution was dialyzed against deionized water for 24 h to obtain DOX-loaded NCMs. DOX_7_-NCM and DOX_10_-NCM refer to the DOX-loaded NCMs with 7 and 10 wt% feed of DOX relative to the polymer, respectively.

Drug loading efficiency (DLE) and drug loading content (DLC) were determined from the below equations by dissolving the definite mass of DOX-loaded micelles in DMSO, and determining the amount of DOX using UV/Vis spectrophotometer at wavelength 490 nm.

$$\mathrm{DLC}\left(\%\right)=\frac{m_{\mathrm{DOX}}}{m_{p}}\;\mathrm{DLE}\left(\%\right)=\frac{m_{\mathrm{DOX}\;}}{m_{\mathrm{DOX}\;in\;the\;feed}}$$

where $m_{\mathrm{DOX}}$ represents the mass of DOX-loaded in the micelle samples, and $m_{p}$ is the mass of DOX-loaded micelles.

### 4.9. Preparation of Blank and DOX-Loaded MeO-PEG-b-PHEMA(ene)-b-PBA CMs

MeO-PEG-*b*-P(HEMA-ene)-*b*-PBA CMs, with different crosslinking densities at the core/shell intermediate layer, were prepared through thiol-ene reaction using 1,4-butanediol bis(3-mercaptopropionate) as crosslinker and DMPA as a photoinitiator. A series of CMs samples were prepared by varying amounts of crosslinker and DMPA in the feed (Table 1). In a typical experiment in which sample CM10 was prepared (the number 10 indicates 10 mol% feed of thiol relative to alkene for crosslinking), stock solutions of polymer (100 mg/mL), crosslinker (8 mg/mL) and DMPA (3 mg/mL) in DMSO were prepared in advance. Then, 100 µL solution of polymer (10 mg, with 2.86 × 10^−5^ mol of alkene, 1.0 equiv), 47.6 µL solution of crosslinker (38.1 × 10^−2^ mg, with 2.86 × 10^−6^ mol of thiol, 0.1 equiv), and 48.6 µL solution of DMPA (14.6 × 10^−2^ mg, 5.71 × 10^−7^ mol, 0.02 equiv) were mixed together, then diluted to 2 mL with DMSO. After that, the solution was added slowly to 3 mL of deionized water and stirred for 3 h at room temperature followed by irradiation with UV light (λ_max_ = 365 nm) for 30 min. The solution was dialyzed against deionized water using dialysis tubing (MWCO = 3500 Da) for 24 h. Similarly, MeO-PEG-*b*-P(HEMA-ene)-PBA CM5 and CM20 were also prepared at 5 and 20 mol% feed of thiol relative to the alkene, respectively. For particle size measurement by DLS, the CM solutions were diluted to 1.0 mg/mL with deionized water.

DOX-loaded MeO-PEG-*b*-P(HEMA-ene)-*b*-PBA CMs were prepared using the aforementioned approach by adding different volumes of neutralized DOX stock solution (equivalent to 10 wt%, relative to the polymer) to the feed. The DLE and DLC were determined as mentioned above. DOX_10_-CM5 and DOX_10_-CM10 represent the DOX-loaded MeO-PEG-*b*-P(HEMA-ene)-*b*-PBA micelles with 10 wt% feed of DOX relative to polymer, crosslinked using 5 and 10 mol% of thiol relative to the alkene, respectively.

### 4.10. Preparation of Targeted cRGD-DOX-CM5

MeO-PEG-*b*-P(HEMA-ene)-*b*-PBA (10 mg), cRGD-SH (1 mg), crosslinker (19.0 × 10^−2^ mg), neutralized DOX (1 mg) and DMPA (14.6 × 10^−2^ mg) in 2 mL of DMSO was added slowly to 3 mL of deionized water, then irradiated with UV light (λ_max_ = 365 nm) for 30 min and dialyzed for 24 h against deionized water to give targeted cRGD- DOX_10_-CM5.

### 4.11. In Vitro pH Sensitive Release of DOX

In vitro release of DOX from DOX-loaded MeO-PEG-*b*-P(HEMA-ene)-*b*-PBA micelles, as either NCMs or CMs, was investigated at two different pHs (5.5 and 7.4) using a dialysis bag (MWCO = 3500 Da) at 37 °C. In each case, 5 mL of DOX-loaded micelle solution was placed in a dialysis bag and immersed in 20 mL of PBS buffer solution (pH 7.4 or 5.5, 0.1 M) with stirring at 150 rpm. At predetermined time intervals, 2 mL of the PBS buffer solution was taken and the same volume of fresh buffer was added to maintain the constant total volume. The concentrations of the released DOX were analyzed using a UV/VIS spectrophotometer at a wavelength of 480 nm. The cumulative drug release $\left(E_{r}\right)$ was determined from the following equation:

$$\;E_{r}\left(\%\right)=\frac{V_{e}\sum_{1}^{n-1}C_{i}+V_{ο}C_{n}}{m_{\mathrm{DOX}}}$$

$m_{\mathrm{DOX}}$ represents the amount of DOX loaded on CM or NCM sample; $V_{ο}$ is the total volume of the release medium (i.e., 20 mL), and *V_e_* is the volume withdrawn (i.e., 2 mL); $C_{n}$ and $C_{i}$ represent the mass/volume concentration of DOX in nth and ith samples, respectively.

### 4.12. Cell Viability Studies

Cytotoxicity of blank or DOX-loaded micelles, as either NCMs or CMs, was assessed against MCF 10A breast cancer cells using MTT colorimetric assay. The cells were seeded at density 5000 cell/well in a 96-well plate with Dulbecco’s Modified Eagle Medium (DMEM) supplemented with 10% FBS. Different concentrations of micelles were added to the cells, then incubated for 24 h under 5% CO_2_ at 37 °C. Subsequently, a solution of MTT reagent dissolved in PBS was added to each well and the plates were incubated for an additional 3 h at 37 °C. Then the medium containing MTT was removed and 150 µL of DMSO was added to each well to dissolve the MTT formazan crystals formed. Finally, the plates were shaken for 10 min and the absorbance was recorded using a plate reader at 490 nm. MTT efficacy was measured by reading the 96-well plate on a microtiter plate reader (Miles Inc., Titertek Multiscan Plus MK II, Huntsville, AL, USA). Cytotoxicity of all samples was calculated as a viability percentage relative to the untreated cells.

### 4.13. In Vitro Cellular Uptake and Imaging

Cellular uptake of DOX, DOX_10_-NCM, DOX_10_-CM5, and cRGD-DOX_10_-CM5 was investigated using MCF 10A tumor and normal cells. Both cells were cultured in 35 mm dishes with essential medium containing FBS (10%) until 60–70% confluence, then incubated with fresh media containing the tested samples of final DOX concentration at 2 μg/mL for 2 h under 5% CO_2_ at 37 °C. After incubation, the cells were washed twice with PBS (pH 7.4) and directly imaged using a laser scanning confocal microscope.

## 5. Conclusions

Well-defined triblock copolymer PEG-*b*-P(HEMA-ene)-*b*-PBA was synthesized through two successive ATRP steps, followed by post-polymerization functionalization of the middle PHEMA block. It was then used as the building unit for the development of micelles, including both NCMs and CMs with tunable crosslinking density at the core/shell intermediate layer. Cumulative DOX release from 5, 10, and 20% crosslinked micelles was investigated, and the results demonstrated their pH-sensitive feature as well as the effective regulation of release rate by adjusting the interfacial crosslinking density. The in vitro MTT assay revealed that the crosslinked micelles are biocompatible to MCF 10A cells, and the drug-loaded samples exhibited a significant therapeutic efficiency as compared to free DOX. cRGD-containing DOX-loaded crosslinked micelles displayed higher cellular uptake relative to their analogues without cRGD. This study demonstrated the synthesis of well-defined PEG-*b*-P(HEMA)-*b*-PBA-based crosslinked micelles with tunable crosslinking density at the core/shell intermediate layer, enabling the regulation of pH-sensitive DOX release. These results indicate that such pH-responsive micelles are therapeutically effective against cancer cells and hold remarkable promise to serve as a smart drug delivery system for cancer therapy.

## Figures and Tables

**Table 1:** Structural parameters of polymers.

	*DP*_n_ for Blocks ^a^			*M*_n,NMR_^a^ (kDa)	*M*_n,GPC_^b^ (kDa)	*Ð* ^b^
	1st	2nd	3rd			
MeO-PEG-Br	44	-	-	2.3	2.2	1.01
PEG-*b*-PHEMA	44	62	-	10.2	10.4	1.15
PEG-*b*-PHEMA-*b*-PBA	44	62	48	16.4	16.7	1.21
PEG-*b*-P(HEMA-ene)-*b*-PBA	44	62	48	21.7	22.0	1.27

^a^ By ^1^H NMR analysis. ^b^ By GPC analysis, relative to polystyrenes.

**Table 2:** Preparation of PEG-*b*-P(HEMA-ene)-*b*-PBA-based micelles with tunable crosslinking density at core-shell interface.

Entry	[ene]_0_/[SH]_0_/[DMPA]_0_	$D_{h,v}\;(\mathrm{nm}){\;}^{b}$	PDI ^b^
NCM	1/0/0	115.5 ± 4.4	0.207
CM5	1/0.05/0.02 ^a^	102.2 ± 2.4	0.265
CM10	1/0.10/0.02 ^a^	90.4 ± 2.3	0.132
CM20	1/0.20/0.02 ^a^	78.8 ± 1.6	0.196

^a^ UV irradiation for 30 min at r.t.; ^b^ By DLS.

**Table 3:** Preparation conditions and characterization of DOX-loaded pH-responsive micelles.

Entry	[ene]_0_/[SH]_0_/[DMPA]_0_^a^	DOX (wt %)	DLC (wt %)	DLE (%)	$D_{h,v}\;(\mathrm{nm}){\;}^{b}$	PDI ^b^
DOX_7_-NCM	-	7.0	6.2	89.6	122.6 ± 4.8	0.283
DOX_10_-NCM	-	10.0	9.1	91.7	128.3 ± 3.4	0.245
DOX_10_-CM5	1/0.05/0.02	10.0	9.7	97.3	106.9 ± 1.1	0.112
DOX_10_-CM10	1/0.10/0.02	10.0	9.5	95.5	95.1 ± 2.4	0.105
cRGD-DOX_10_-CM5	1/0.10/0.02	10.0	9.4	94.8	135.5 ± 4.4	0.248

^a^ UV irradiation for 30 min at r.t.; ^b^ By DLS

## Data Availability

The Supporting Information is available free of charge on the MDPI Publications website.

## Supplementary Materials

The following supporting information can be downloaded at: https://www.mdpi.com/article/10.3390/molecules27238153/s1, Figure S1: CMC analysis of PEG-*b*-P(HEMA-ene)-*b*-PBA; Figure S2: TEM images of CM10 and CM20.

## Abbreviations

CMC, critical micelle concentration; CMs, crosslinked micelles; cRGD, cyclo(Arg-Gly-Asp-D-Phe-Cys); DOX, doxorubicin; EPR, enhanced permeability and retention; non-crosslinked micelles (NCMs); PEG, polyethylene glycol; PEG-*b*-PHEMA-*b*-PBA, poly(ethylene glycol)-*b*-poly(2-hydroxyethyl methacrylate)-*b*-poly(butyl acrylate); NCs, nanocapsules; NPs, nanoparticles; ROP, ring-opening polymerization; NMR, nuclear magnetic resonance; GPC, gel permeation chromatography; NTA, nanoparticle tracking analysis; TEM, transmission electron microscopy.
