# Supplementary material for: Well-Defined pH-Sensitive Self-Assembled Triblock Copolymer-Based Crosslinked Micelles for Efficient Cancer Chemotherapy
Source: Molecules. 2022 Nov 23;27(23):8153. doi: 10.3390/molecules27238153 (PMC9735831; doi:10.3390/molecules27238153)
Supplement: Supplementary file 1 [file molecules-27-08153-s001.zip › molecules-2033456-supplementary.pdf]

molecules

Supplementary Materials

Well-Defined pH-Sensitive Self-Assembled Triblock Copolymer-Based Crosslinked Micelles for Efficient Cancer Chemotherapy

Mohamed Alaa Mohamed [1,2,*], Ajay Singh [3], Paras N. Prasad [3] and Chong Cheng [1]

1 Department of Chemical and Biological Engineering, University at Buffalo, The State University of New York, Buffalo, NY 14260, USA
2 Chemistry Department, Faculty of Science, Mansoura University, Mansoura 35516, Egypt
3 Institute for Lasers, Photonics and Biophotonics, Department of Chemistry, University at Buffalo, The State University of New York, Buffalo, NY 14260, USA
* Correspondence: mm446@buffalo.edu

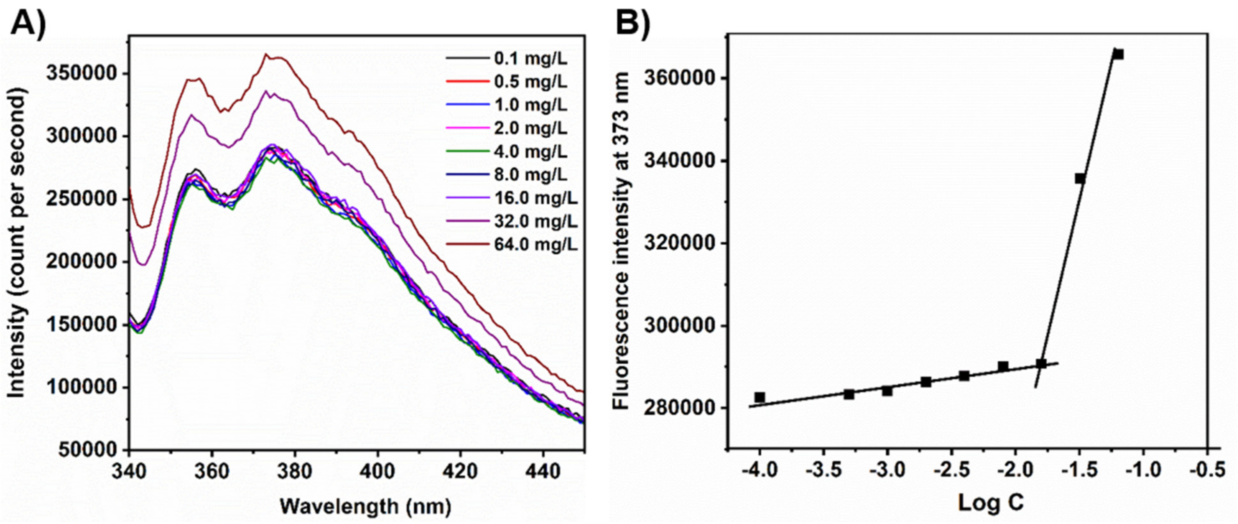

Figure S1. (**A**) fluorescence spectra of pyrene at different polymer concentrations, and (**B**) Log C versus I $_{373}$ plot of pyrene.

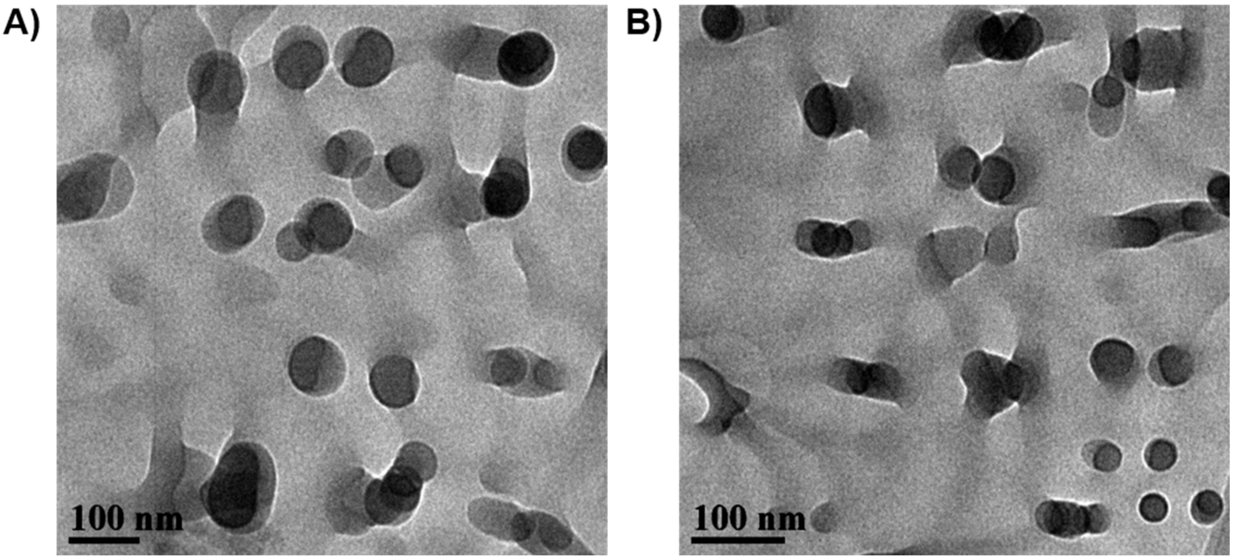

Figure S2. TEM images of (**A**) CM10 and (**B**) CM20.
